# Molecular Characterization of LKB1 of Triploid Crucian Carp and Its Regulation on Muscle Growth and Quality

**DOI:** 10.3390/ani12182474

**Published:** 2022-09-19

**Authors:** Anli Zuo, Yonghua Zhou, Yingjie Li, Yu Zhang, Zilin Yi, Yangbo Xiao, Mei Zou, Shenping Cao, Fufa Qu, Jianzhou Tang, Zhen Liu

**Affiliations:** 1 College of Life Sciences, Hunan Normal University, Changsha 410081, China; 2 Hunan Provincial Key Laboratory of Nutrition and Quality Control of Aquatic Animals, Changsha University, Changsha 410022, China

**Keywords:** LKB1, molecular characterization, muscle growth, muscle quality, myogenic factors, triploid crucian carp

## Abstract

**Simple Summary:**

Liver Kinase B1 (LKB1) can regulate energy metabolism and skeletal muscle growth. In the study, we cloned LKB1 cDNA and assessed LKB1 expression in triploid crucian carp (*Carassius auratus*), the molecular regulation mechanisms of LKB1 involved in muscle growth and quality. In conclusion, the LKB1 amino acid sequence of the triploid crucian carp had a high sequence identity with carp (*Cyprinus carpio*). Additionally, activated LKB1 can promote the expression of most myogenic regulatory factors, muscle fibers development, the formation of inosine monophosphate (IMP), which is a flavor substance of muscle, and improve meat quality by increasing elasticity and chewiness. Inhibiting LKB1 can reverse the trend. Moreover, with increasing lysine-glutamate dipeptide concentrations in the feed, the expression of LKB1 gradually increased and was highest when dipeptide concentration was 1.6%.

**Abstract:**

Liver Kinase B1 (LKB1) is a serine/threonine kinase that can regulate energy metabolism and skeletal muscle growth. In the present study, LKB1 cDNA of triploid crucian carp (*Carassius auratus*) was cloned. The cDNA contains a complete open reading frame (ORF), with a length of 1326 bp, encoding 442 amino acids. Phylogenetic tree analysis showed that the LKB1 amino acid sequence of the triploid crucian carp had a high sequence similarity and identity with carp (*Cyprinus carpio*). Tissue expression analysis revealed that LKB1 was widely expressed in various tissues. LKB1 expressions in the brain were highest, followed by kidney and muscle. In the short-term LKB1 activator and inhibitor injection experiment, when LKB1 was activated for 72 h, expressions of myogenic differentiation (MyoD), muscle regulatory factor (MRF4), myogenic factor (MyoG) and myostatin 1 (MSTN1) were markedly elevated and the content of inosine monophosphate (IMP) in muscle was significantly increased. When LKB1 was inhibited for 72 h, expressions of MyoD, MyoG, MRF4 and MSTN1 were markedly decreased. The long-term injection experiment of the LKB1 activator revealed that, when LKB1 was activated for 15 days, its muscle fibers were significantly larger and tighter than the control group. In texture profile analysis, it showed smaller hardness and adhesion, greater elasticity and chewiness. Contrastingly, when LKB1 was inhibited for 9 days, its muscle fibers were significantly smaller, while the gap between muscle fibers was significantly larger. Texture profile analysis showed that adhesion was significantly higher than the control group. A feeding trial on triploid crucian carp showed that with dietary lysine-glutamate dipeptide concentration increasing, the expression of the LKB1 gene gradually increased and was highest when dipeptide concentration was 1.6%. These findings may provide new insights into the effects of LKB1 on fish skeletal muscle growth and muscle quality, and will provide a potential application value in improvement of aquaculture feed formula.

## 1. Introduction

Fish is an important protein source for humans. The essence of aquaculture is to promote the rapid muscle growth and quality and increase the efficiency of aquaculture production. Muscle growth is regulated by myogenic regulatory factors (MRFs), including myogenic differentiation (MyoD), myogenic factor 5 (Myf5), muscle regulatory factor (MRF4) and myogenic factor (MyoG) [1,2,3]. When MRFs are activated, they induce myoblast differentiation by regulating the transcription of many coding and non-coding genes to form specific heterodimers with synergistic regulatory factors, forming fibers across the sarcomere length [4,5]. Myostatin, a transforming growth factor-β (TGF-β) member, is a negative regulator of muscles that is mainly expressed and secreted on skeletal muscles [6]. While modern aquaculture is aimed at ensuring high yields and high efficiency, meat quality and flavor of aquatic animals are sharply declining. Therefore, there is a need to improve the yields and muscle quality to improve the flavor of cultured fish. Inosine monophosphate (IMP) is the secondary metabolite of adenosine triphosphate (ATP) [7] and is also the main source of meat flavor. In living cells, ATP is continuously synthesized and decomposed, while IMP content maintains at a low level [8]. In meat, IMP is mainly derived from ATP degradation during slaughter and postmortem aging [9]. After slaughter, ATP synthesis in animals is interrupted, and most ATP is decomposed into adenosine diphosphate (ADP) under the actions of various enzymes, and then into adenosine monophosphate (AMP) and IMP. Given that the generated IMP is unstable, it decomposes into a bitter inosine [10]. The effect of IMP on meat flavor is 50 times that of sodium glutamate [11,12]. Therefore, IMP is an important index for evaluating meat flavor [13].

Liver kinase B1 (LKB1), also known as STK11 (serine threonine kinase 11), is a serine/threonine kinase that was originally found in Peutz Jeghers syndrome (PJs) [14]. LKB1 is a tumor suppressor that plays an important role in promoting skeletal muscle development [15] and regulating energy metabolism [16]. Biologically, LKB1 knockout promotes muscle satellite cell proliferation and inhibits myoblast differentiation, which significantly down regulates myogenic factor expressions and reduces muscle regeneration abilities [17]. LKB1, the most important upstream kinase of Adenosine 5′-monophosphate activated protein kinase (AMPK), can activate AMPK expressions [17]. As a cell fuel sensor, AMPK inhibits the anabolic process of ATP consumption, opens the nutritional catabolic pathway and generates ATP [18]. Therefore, LKB1 activation leads to accumulation of more ATP in the body. Currently, studies on LKB1 have majorly focused on the fields of pathology, physiology, immunity and nerves. It is necessary to investigate the effects of LKB1 on fish growth, development and meat quality. 

Amino acids are essential elements for maintaining animal growth and health. Given that fish cannot fully synthesize all protein-derived amino acids, they must be obtained from diets [19]. Glutamate plays a crucial regulatory role in immunity, metabolism, growth and in meat quality control [20,21]. Lysine is a restricted essential amino acid in many plant proteins. Lack of lysine in fish usually leads to growth retardation [10,22,23]. Therefore, supplementation of appropriate levels of glutamate and lysine to fish diet is of great significance for improving feed formula.

Crucian carp, a freshwater fish, is one of the important edible fish in China with a tender and nutritious meat. Triploid crucian carp (3n = 150) is characterized by fast growth rates, sterility and good meat quality. It is cultivated on a large scale in 26 provinces and cities in China [24]. The molecular mechanisms involved in nutritional regulation in triploid crucian carp have not been fully established. Therefore, we cloned LKB1 cDNA and assessed the expression of LKB1 in triploid crucian carp and the molecular regulation mechanism involved in growth and development, as well as meat quality and flavor, which were also evaluated for the first time. The finding revealed the roles of LKB1 in regulation of muscle development and meat quality of teleost fish, thereby contributing to the improvement of fish feed formula in aquaculture.

## 2. Materials and Methods

### 2.1. Animals and Tissue Preparation

All triploid crucian carp in this study were provided by the Hunan Institute of Aquatic Sciences, and all of them were anesthetized with 20 mg/L MS-222 (Sigma, Alcobendas, Spain) and sacrificed before examination. Tissue samples were collected from the intestines, heart, liver, brain, muscles, as well as gills and stored at −80 °C for further analyses. 

### 2.2. Cloning of the cDNA Sequence of LKB1

LKB1 sequences of the *Cyprinidae* species were directly searched in the NCBI database after which their cDNA sequences were amplified by reverse transcription–polymerase chain reaction (RT-PCR). Primers specific for each gene were designed by the Premier 5.0 program according to the conserved gene sequence (Table 1). The PCR template was synthesized from 1.0 μg of the muscular RNA of triploid crucian carp using a PrimeScript™ 1st Strand cDNA Synthesis Kit (Takara, Kyoto, Japan), as instructed by the manufacturer. PCR amplification was performed in a total reaction volume of 25.0 μL containing 2.5 μL 10 × LA PCR Buffer II (Mg_2_^+^ plus), 1.0 μL cDNA temple, 4.0 μL dNTP mixture (2.5 mM each), 1.0 μL of each primer (10.0 μM), 15.25 μL ddH_2_O, and 0.25 μL LA-Taq DNA Poly-merase (TaKaRa, Japan). The PCR conditions were: 94 °C for 2 min, 35 cycles of 30 s at 94 °C, 30 s at 55 °C and 2 min at 72 °C, followed by a final extension for 10 min at 72 °C. PCR products were analyzed by 1.0% agarose gel/TAE electrophoresis and purified using a HiPure Gel Pure Micro Kit (Magen, Guangzhou, China). Then, PCR products were sequenced on a 3730 Applied Biosystems (ABI) DNA sequencer and verified by the BLAST program (http://blast.ncbi.nlm.nih.gov/Blast.cgi) (accessed on 12 October 2021).

### 2.3. Phylogenetic Analysis of LKB1

The phylogenetic tree was constructed using the neighbor-joining method and LKB1 homolog sequences from triploid crucian carp and other vertebrates. Amino acid sequences were analyzed using the NCBI online program, ORF finder. In MEGA 11, 1000 bootstrap repetitions were used to assess the reliability of the phylogenetic tree. 

### 2.4. LKB1 Expressions in Various Tissues 

For tissue distribution analysis of LKB1 mRNA, total RNA was isolated from eight different tissues (muscle, heart, spleen, intestines, gills, brain, liver and kidney) of the triploid crucian carp. The RNA samples were isolated from all triploid crucian carp tissues and used for cDNA synthesis. Then, mRNA expressions of LKB1 were analyzed by Quantitative Real-time PCR (qRT-PCR). In this context, relative expression levels of all LKB1 mRNAs were derived from the results of β-actin.

### 2.5. Intraperitoneal Injection of the Activator and Inhibitor

Triploid crucian carp (12.0 ± 0.5) g were randomized into four groups for LKB1 specific activator Gomisin J (MedChemExpress, Monmouth Junction, NJ, USA, CAS No. 1093222-27-5) and inhibitor Pim1/AKK1-IN-1 (Pim) (MedChemExpress, CAS No. 66280-25-9) injection experiments. The experiment was performed by injecting 100 µL of phosphate- 222 buffered saline (PBS) to the activator control group 200 µL of PBS to the inhibitor control group, injecting 100 µL Gomisin J at doses of 10 mg/kg to the experimental group 1, and 200 µL Pim at doses of 40 mg/kg to the experimental group 2. Three repetitions in each group were performed and each repetition had 4 fish. 

In short-term injection experiments, at 24 and 72 h after injection, all samples (muscles) were collected, after which relative mRNA expressions of LKB1 and myogenic factors (MyoG, MyoD, MRF4, Myf5, MSTN1) were detected. Moreover, IMP levels were determined. 

In long-term injection experiments, injections were performed every 72 h. At 9 days after injection, muscle samples of the inhibitor control group and the experimental group 2 were collected for paraffin section and meat quality analysis. At 15 days after injection, muscle samples of the activator control group and the experimental group 1 were collected for paraffin section and meat quality analysis.

### 2.6. Determination of IMP Levels

In this assay, 0.02, 0.04, 0.06, 0.08 and 0.1 mg/mL IMP standard solutions were prepared. Then, at 72 h after injection, 0.6 g muscle was measured in a beaker containing 40 mL 95 °C water, which was then put into a 95 °C constant-temperature water bath, cooked for 5 min and cooled to room temperature and constant volume (50 mL). The solution was filtered via a filter membrane and sampled for analysis. An external standard method was used for quantitative analysis. The high-performance liquid chromatography was performed on a CBM-10A VP Plus (Shimadzu, Kyoto, Japan). The chromatographic column was an Agilent Zorbax sb-c18 column. The mobile phase was methanol: 50 mmol/L ammonium acetate aqueous solution (5:95, V/V). The flow rate was 1.0 mL/min. The injection volume was 10 μL and the detection wavelength was 254 nm. 

### 2.7. Preparation of Paraffin Sections of Muscle Fibers

Muscles were fixed in paraformaldehyde, washed, dehydrated, waxed, embedded in paraffin and sliced into 6 μM thick sections. Paraffin sections were stained with hematoxylin and eosin (HE) and sealed using a neutral resin. Then, they were observed under an inverted microscope. Areas with clear staining and no impurities or obvious cell contours were selected to obtain 200× images and 400× images.

### 2.8. Measurement of Muscle Fiber Diameter and Area

After muscle sections had been stained with HE, 5 visual fields of 400× were randomly selected from each section. The longest Feret diameter and shortest Feret diameter of 100 muscle fibers were measured using the Image J software (National Institutes of Health, Bethesda, MD, USA, version 1.52p) (muscle fibers were selected in muscle bundles, and all muscle fibers in each muscle bundle were completely measured). The geometric mean value was calculated.

### 2.9. Detection of Muscle Texture Characteristics

Middle back muscles in different treatment groups were sliced into 0.5 cm × 0.5 cm × 0.5 cm fish blocks. Hardness, adhesiveness, cohesiveness, springiness, gumminess and chewability of muscles were assessed by TMS Pro texture profile analysis (TPA). Parameter settings were: the range of force sensing element was 250 N, the detection speed was 30 mm/min, shape variability was 60% and the starting force was 0.1 N. 

### 2.10. Effects of Dietary Lysine-Glutamate Dipeptides on LKB1 Expressions

The effects of six levels of lysine-glutamate dipeptides (0, 0.4%, 0.8%, 1.2%, 1.6% and 2.0%) (Table 2) on LKB1 gene expressions were assessed. All the fish were fed for two weeks to adapt to the experimental feed and environment. About 450 healthy triploid crucian carp (11.79 ± 0.09 g) were selected and randomized into 6 groups with 3 repetitions in each group and each repetition had 25 fish. The triploid crucian carp were fed twice a day. After 8 weeks of the feeding test, feeding was stopped for 1 day, after which fish in each group were uniformly sampled (*n* = 3 for each group) for assessment of LKB1 expressions in the muscles by qRT-PCR.

### 2.11. Statistical Analysis

Data are expressed as mean ± SEM. Differences in mean were assessed by one-way analysis of variance (ANOVA) followed by the Duncan’s multiple comparison tests. The SPSS 18 software was used for statistical analysis. *p* < 0.05 was set as the threshold for statistical significance.

## 3. Results

### 3.1. Isolation and Sequence Analysis of LKB1 cDNA

**Figure 1:**
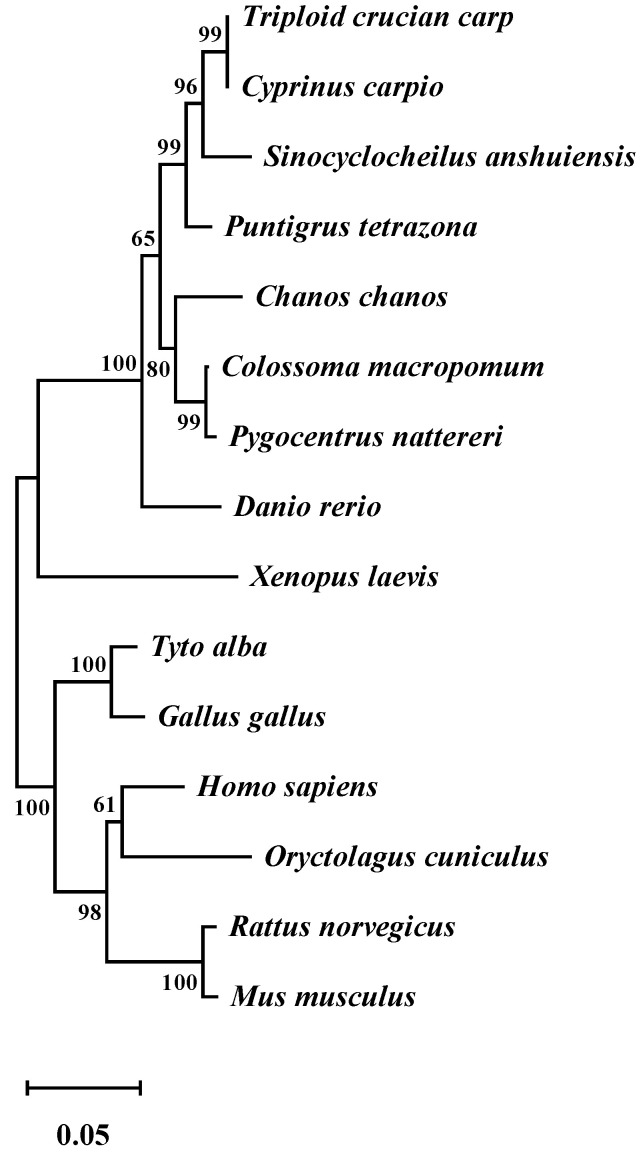
Phylogenetic tree of LKB1 constructed using the Neighbor-Joining method. The accession numbers are as below: *triploid crucian carp* OL961664, *Cyprinus carpio* XP_042575073.1, *Puntigrus tetrazona* XP_043119227.1, *Danio rerio* NP_001017839.1, *Sinocyclocheilus anshuiensis* XP_016359770.1, *Chanos chanos* XP_030631382.1, *Colossoma macropomum* XP_036412081.1, *Pygocentrus nattereri* XP_017552678.1, *Xenopus laevis* NP_001083758.1, *Homo sapiens* NP_000446.1, *Tyto alba* XP_032849343.1, *Gallus gallus* NP_001039298.1, *Oryctolagus cuniculus* AMB66545.1, *Rattus norvegicus* NP_001101539.1, *Mus musculus* BAA76749.1. Numbers at the nodes represent the percentages of 1000 bootstrap replicates.

The cDNA of LKB1 from the muscle was used to establish a cDNA library of triploid crucian carp. The sequence was uploaded to the GenBank database (GenBank accession number is OL961664). Using Editseq software (DNASTAR, Madison, WI, USA, version 7.1.0), it was established that the total length of cDNA was 1348 bp, including a complete open reading frame (ORF) sequence, with a total length of 1326 bp, encoding 442 amino acid residues. The evolutionary tree in Figure 1 showed that LKB1 can be divided into two branches: one was the branch of bony fish, while the other was the branch of birds and mammals. LKB1 of the triploid crucian carp exhibited a high sequence similarity and identity with LKB1 of *Cyprinus carpio*, implying that the functions of LKB1 in the two species of fish were similar.

### 3.2. Tissue Expression Patterns of LKB1

**Figure 2:**
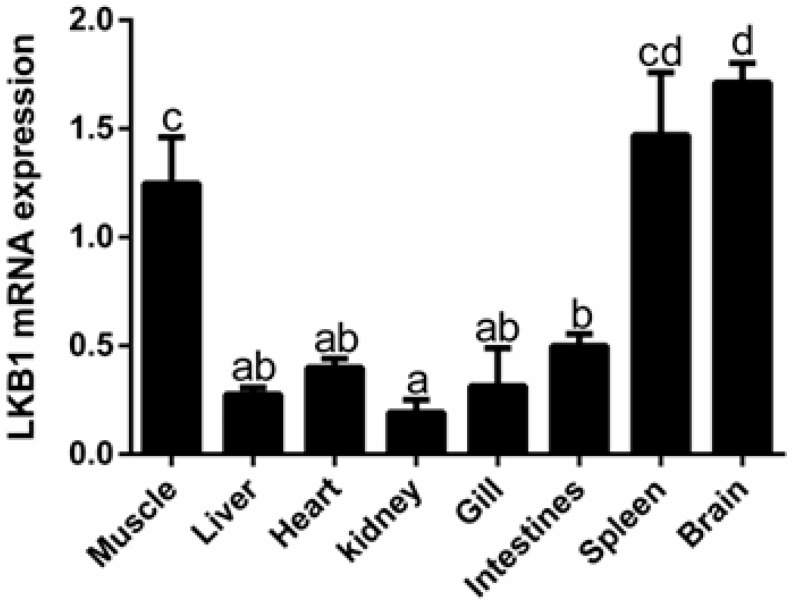
Relative mRNA expression levels of LKB1 in different tissues. Data are presented as means ± SE (*n* = 3). Different letters indicate significant differences (*p* < 0.05).

LKB1 was expressed in all tissues, with its expressions in the brain being the highest, followed by the spleen and muscle (Figure 2). Expressions in the kidney were lowest. 

### 3.3. Effect of LKB1 Activator or Inhibitor on Triploid Crucian Carp Muscle Growth

**Figure 3:**
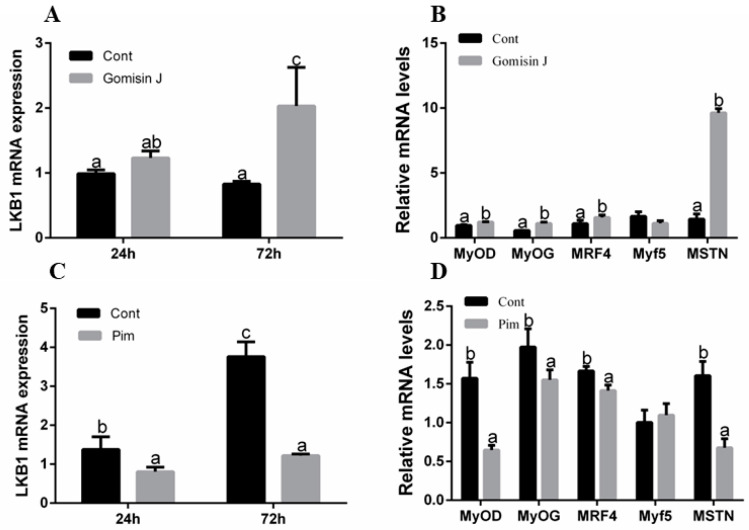
The effects of Gomisin J and Pim on triploid crucian carp muscular LKB1 and myogenic factor expression at different time points. (**A**) The effects of Gomisin J on LKB1 expression at different time points. (**B**) The effects of Gomisin J on MyoD, MyoG, MRF4, Myf5 and MSTN1 expression at 72 h. (**C**) The effects of Pim on LKB1 expression at different time points. (**D**) The effects of Pim on myogenic factor (MyoD, MyoG, MRF4, Myf5 and MSTN1) expression at 72 h. Data are presented as means ± SE (*n* = 3). Different letters indicate significant differences (*p* < 0.05).

Through the activator and inhibitor experiments of LKB1, the changes of myogenic factors and muscle fiber development were investigated. The effects of Gomisin J on expressions of LKB1 and myogenic factors are shown in Figure 3A,B. Figure 3A shows that LKB1 mRNA expressions in the Gomisin J group at two time points were higher, relative to the control group, with significant differences at 72 h (*p* < 0.05). Therefore, myogenic factor expressions in control and activator groups were determined at 72 h. Figure 3B shows that, at 72 h, mRNA expressions of MyoG, MyoD, MRF4 and MSTN1 in the Gomisin J group were significantly high relative to control group (*p* < 0.05). Expressions of Myf5 between the groups were insignificant (*p* > 0.05). The effects of Pim on expressions of LKB1 and myogenic factors are shown in Figure 3C,D. Figure 3C shows that, compared to the control group, at 24 and 72 h, LKB1 mRNA expressions in the Pim group were significantly low (*p* < 0.05), and the trend was most obvious at 72 h. Therefore, we measured myogenic factor expressions in control and inhibitor groups at 72 h. Figure 3D shows that, at 72 h, MyoG, MyoD, MRF4 and MSTN1 expressions in the Pim group were significantly suppressed relative to the control group (*p* < 0.05), and differences in expressions of Myf5 between the groups were insignificant (*p* > 0.05).

**Figure 4:**
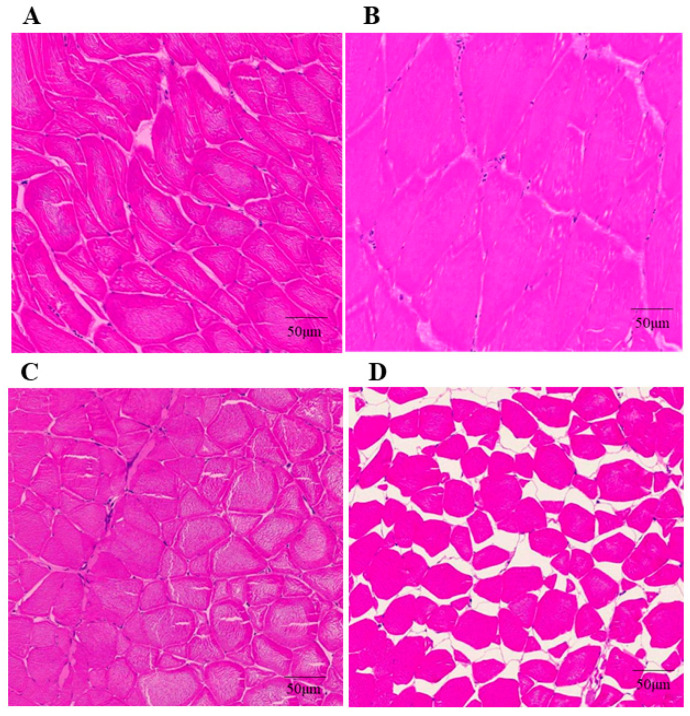
The effects of Gomisin J and Pim on triploid crucian carp histological characteristics of muscle fibers. (**A**) H&E staining (original magnification × 200) exhibits a distribution of muscle fiber and muscle fiber space 15 days after PBS injection. (**B**) H&E staining (original magnification × 200) exhibits a distribution of muscle fiber and muscle fiber space 15 days after Gomisin J injection. (**C**) H&E staining (original magnification × 200) exhibits a distribution of muscle fiber and muscle fiber space 9 days after PBS injection. (**D**) H&E staining (original magnification × 200) exhibits a distribution of muscle fiber and muscle fiber space 9 days after Pim injection.

The effects of Gomisin J and Pim on histological properties of muscle fibers are shown in Figure 4. Figure 4A,B shows cross sections of skeletal muscle fibers in the control group and the Gomisin J group exhibited irregular polygonal shapes, without significant differences in morphology. Figure 4C,D shows that differences in morphologies of muscle fibers between the control and Pim groups were insignificant. Compared to the control group, muscle fibers in the Pim group were significantly shrunk and the gap between muscle fibers increased.

**Figure 5:**
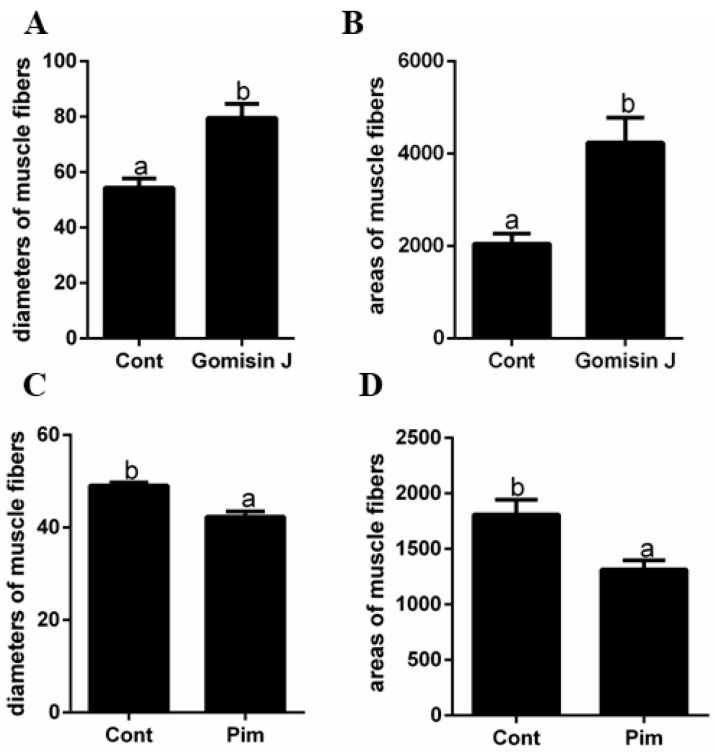
The effects of Pim on triploid crucian carp histological characteristics of muscle fibers. (**A**) The effects of Gomisin J on diameters of muscle fibers at 15 days. (**B**) The effects of Gomisin J on areas of muscle fibers at 15 days. (**C**) The effects of Pim on diameters of muscle fibers at 9 days. (**D**) The effects of Pim on areas of muscle fibers at 9 days. Data are presented as means ± SE (*n* = 3). Different letters indicate significant differences (*p* < 0.05).

Quantitative analysis of different positional muscle fiber sections is shown in Figure 5. The average muscle fiber diameter and area in the Gomisin J group were significantly high relative to those of the control group (*p* < 0.05), implying that Gomisin J promoted skeletal muscle fiber development in triploid crucian carp (Figure 5A,B). Compared to the control group, the average muscle fiber diameter and area in the Pim group were significantly low relative to control group (*p* < 0.05), indicating that Pim inhibits muscle fiber development (Figure 5C,D).

### 3.4. Effect of LKB1 Activator and Inhibitor on Triploid Crucian Carp Muscle Quality

**Figure 6:**
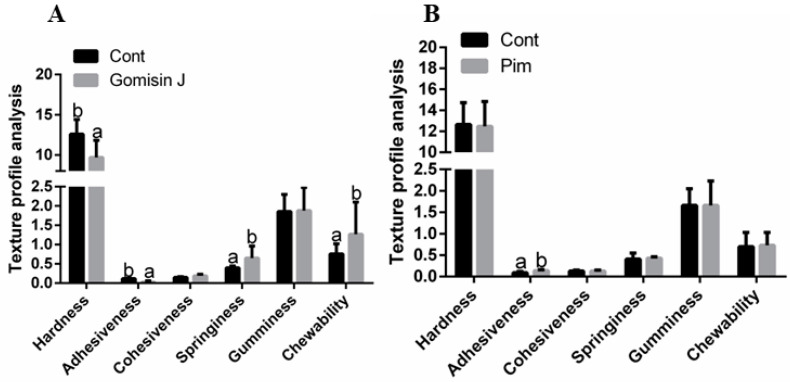
The effects of Gomisin J and Pim on triploid crucian carp muscular texture features at different time points. (**A**) The effects of Gomisin J on muscular texture features at 15 days. (**B**) The effects of Pim on muscular texture features at 9 days. Data are presented as means ± SE (*n* = 3). Different letters indicate significant differences (*p* < 0.05).

Hardness and adhesiveness of muscles from the Gomisin J group were significantly low relative to control group (*p* < 0.05), while springiness and chewability were significantly high (*p* < 0.05). Differences in cohesiveness and gumminess were insignificant (*p* > 0.05). Adhesiveness of the Pim group was significantly high relative to control group (*p* < 0.05), however, differences in hardness, cohesiveness, springiness, gumminess and chewability between the groups were insignificant (*p* > 0.05) (Figure 6).

**Figure 7:**
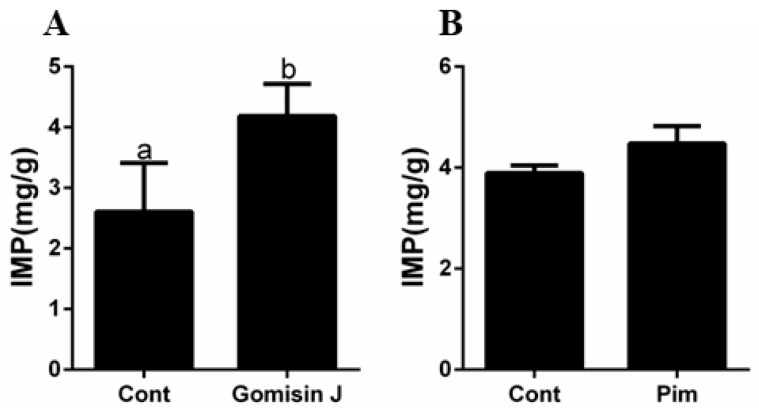
The effects of Gomisin J and Pim on triploid crucian carp flavoring substance inosine monophosphate (IMP) at different time points. (**A**) The effects of Gomisin J on IMP content at 72 h. (**B**) The effects of Pim on IMP content at 72 h. Data are presented as means ± SE (*n* = 3). Different letters indicate significant differences (*p* < 0.05).

The effects of Gomisin J and Pim on IMP levels are shown in Figure 7. Compared to control group, IMP levels in the Gomisin J group were markedly increased (*p* < 0.05), while IMP levels between the Pim and control group at 72 h were not marked (*p* > 0.05).

### 3.5. The Effects of Lysine-Glutamate Dipeptide on the Relative mRNA Expression of LKB1 in Muscle

**Figure 8:**
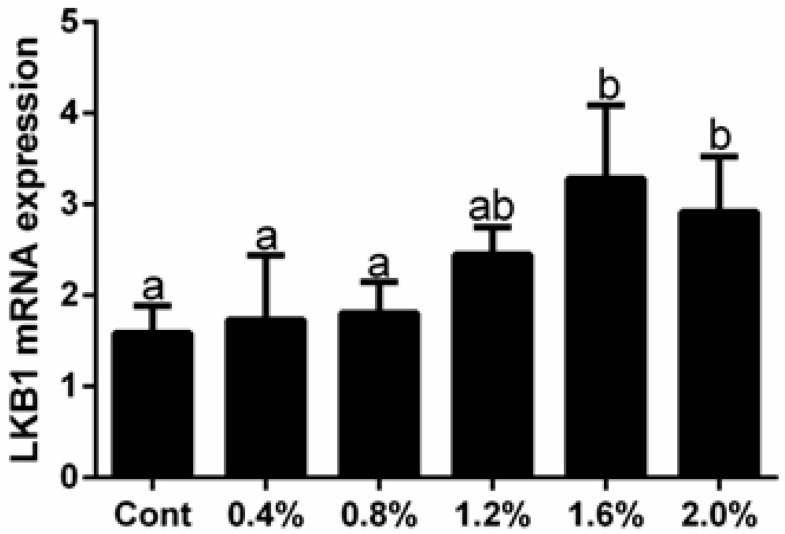
The effects of lysine-glutamate dipeptide diets with different concentrations on LKB1 expression. Different percentages represent different concentrations of dipeptide. Data are presented as means ± SE (*n* = 3). Different letters indicate significant differences (*p* < 0.05).

The effects of lysine-glutamate dipeptide diets with different concentrations on LKB1 expressions in muscle are shown in Figure 8. Compared to control group, with increasing dipeptide concentrations in the feed, expression levels of LKB1 were gradually increased, and were highest at dipeptide concentrations of 1.6% (*p* < 0.05). This indicated that high concentrations of the dipeptide diet promoted LKB1 expressions.

## 4. Discussion

LKB1 is an important kinase that is involved in regulating life activities, including energy metabolism [25,26], tumorigenesis [27], skeletal muscle growth and development [28]. In this study, the LKB1 sequence of triploid crucian carp was first cloned. By comparing the amino acid sequences encoded by LKB1 of different species and constructing the genetic evolution tree between different species, we can preliminarily judge the genetic relationship of different species at the micro level. Comparisons of LKB1 sequences of different species revealed that triploid crucian carp belongs to the branch of bony fish, which is in the same branch as *Cyprinus carpio*. The homology of the ORF region of LKB1 is highest, gene function similarities are highest, and genetic relationships are closest. The fish with closest genetic relationships with triploid crucian carp also include *Sinocyclocheilus anshuiensis* and *Puntigrus tetrazona*. *Rattus norvegicus* and *Mus musculus* were the farthest to be related to triploid crucian carp, which conforms to the law of biological evolution.

LKB1 is widely expressed in all tissues [29]. The absence of LKB1 blocks LKB1/AMPK signal transduction, resulting in abnormal activation of mTOR, thereby promoting tumorigenesis. In humans, the absence of LKB1 in different tissues results in colorectal cancer, breast cancer, lung cancer, liver cancer and other cancers, indicating that normal expressions of LKB1 in various human tissues is very important [30]. Studies on LKB1 mutant mice have shown that LKB1 deletion in lungs leads to adenocarcinoma formation [27], LKB1 deletion in skeletal muscles leads to glucose uptake defects as well as growth and development defects [17], and liver-specific LKB1 deletion leads to metabolic defects [31]. In zebrafish (*Danio rerio*), the LKB1 mutant larvae suddenly lost intestinal folding abilities at 7 days post fertilization, and premature consumption of glycogen in the liver resulted in increased liver lipid accumulation, indicating that normal expressions of LKB1 are essential for early organ development in zebrafish [32]. In this study, the LKB1 gene was found to be generally expressed in various tissues, implying that it is involved in many physiological processes in triploid crucian carp. LKB1 is mainly expressed in the brain, spleen and muscles, which indicates that it is necessary in these tissues [17,33], laying the basis for assessment of regulatory mechanisms of LKB1 on muscle development.

Skeletal muscle development is regulated by a series of myogenic regulatory factors [1,3]. MRFs play a crucial role in orientation and differentiation of skeletal muscles at the embryonic stage and after birth [3]. In this study, when LKB1 was activated for 72 h, expressions of MyoD, MRF4 and MyoG in muscles were markedly increased. In contrast, when LKB1 was inhibited, MyoD, MRF4 and MyoG expressions in muscles were markedly suppressed. This suggests that LKB1 has a positive regulatory role on MRFs. The second key regulator of muscle growth is MSTN1. Fish study has shown that MSTN1 negatively regulates skeletal muscle growth [34]. However, negative regulation of MSTN1 on skeletal muscle growth and development is still controversial. Growth hormone receptors-1/-2 (GHRs) are growth hormone receptors, which play an important role in stimulating muscle protein synthesis. A significant positive relationship between MSTNs and GHRs expression in blunt snout bream (*Megalobrama amblycephala*) was observed in a previous study [35]. This implies that MSTN is not simply a muscle growth inhibitor, the relationships between GHRs and MSTNs were more complex than expected. Differences in outcomes between these studies remain to be clarified. We established that when LKB1 was activated for 72 h, myoblast differentiation gene and MSTN1 expressions were significantly elevated, suggesting that LKB1 may enhance the expression of the myoblast differentiation gene and promotes muscle development by upregulating MSTN1 levels. Comparatively, Garikipati and Rodgers showed that with increasing MSTN1 expressions, expressions of myoblast differentiation marker genes (Myf5, MyoD and MyoG) in rainbow trout (*Oncorhynchus mykiss* stationary) muscle satellite cells were increased [36]. This is similar to our results. Further assays revealed that MSTN1 initiated differentiation by inhibiting muscle satellite cell proliferations [37]. These studies suggest that describing the effects of MSTN1 as pure inhibition may be too simple, because it seems to promote cell differentiation by inhibiting the proliferation of primary muscle satellite cell differentiation, especially in fish. Histochemical analysis of muscle fibers after LKB1 activation for 15 days or LKB1 inhibition for 9 days was performed. Compared to the control group, muscle fibers in the Gomisin J group were significantly more hypertrophic, as verified by muscle fiber areas and diameters. In contrast, compared to control group, muscle fibers in the Pim group shrunk significantly and the gap between muscle fibers was increased. Further analysis revealed that the average muscle fiber diameter and area in the Pim group were significantly low relative to control group. Kwasek et al. showed that MyoD and MyoG expressions in muscles of fast-growing Yellow Perch (*Diploprion bifasciatum*) were higher relative to slow-growing Yellow Perch. Muscle fiber areas for fast-growing Yellow Perch were also larger [38]. Therefore, we postulated that LKB1 could promote muscle growth of triploid crucian carp by regulating the expressions of MRFs and MSTN1. Shan et al. reported that LKB1 deletion could promote muscle satellite cell proliferation and self-renewal, inhibit myogenic differentiation of skeletal muscle progenitor cells, and affect skeletal muscle growth as well as development [15]. It was found that LKB1-knockdown mice developed severe myopathy, characterized by severe muscular dystrophy, growth retardation and premature death. In addition, satellite cells with LKB1 deletion automatically lose the ability to regenerate. Similarly, Thomson found that mice with skeletal muscle and myocardial LKB1 double knockout had skeletal muscle atrophy and severe atrial dilation, suggesting cardiac insufficiency and heart failure [33]. On the one hand, LKB1 regulates muscle satellite cell proliferation via the classical AMPK/mTOR pathway, on the other hand, LKB1 is at least partially independent of AMPK and, through the GSK-3β pathway, regulates myoblast differentiation during muscle development [28,30]. These findings confirm that LKB1 is a key regulator of myoblast proliferation and differentiation, and establishes the core role of LKB1 in muscle stem cell homeostasis, muscle development and regeneration [28].

IMP is an important indicator for evaluating meat flavor [13]. In this study, when LKB1 was activated for 72 h, IMP levels in triploid crucian carp were significantly elevated. This could have been because LKB1 affected IMP formation by regulating energy metabolism. After fish had been killed, ATP decomposes and produces more IMP. Sakamoto et al. found that the AMP:ATP ratio in skeletal muscles of LKB1 knockout mice was significantly elevated, indicating that when LKB1 activity is lost, muscle cells cannot overcome energy imbalances by producing ATP [39]. However, we found that when LKB1 was inhibited for 72 h, differences in IMP levels between Pim and the control group were insignificant. We postulated that this may be because the LKB1-AMPK signaling pathway is not the only way to regulate energy metabolism. The fish also supplied energy through other energy supply pathways, so that the final metabolite IMP was not significantly different from the control group. Therefore, further investigations are required to elucidate LKB1-regulated energy metabolism through another signaling pathway. Texture is an important factor affecting fish meat quality, and muscle fiber diameter and density are important factors affecting muscle texture [40,41]. Generally, the higher the density and smaller the diameter of the muscle fiber, the higher the hardness of the muscle [42,43]. In our 15-day LKB1 activator injection experiment, the fish fillets with the lowest hardness were from the Gomisin J group, and their muscle was characterized by the largest muscle fiber diameter and area. In the 9-day LKB1 inhibitor injection experiment, there were no significant differences in hardness between the control and experimental groups, which may have been due to insufficient injection time.

As one of the final products of protein digestion and hydrolysis in fish intestines, compared to free amino acids, adding appropriate amounts of small peptides to the diet can improve the digestibility of animals and promote their growth and development [19,44]. In this study, we supplemented feeds with lysine-Glutamate dipeptide to evaluate the effects of different concentrations of dipeptide on LKB1 expressions in muscles. We found that there were no significant changes in LKB1 expressions when dipeptide concentrations were low. With increasing dipeptide concentrations, expressions of LKB1 were significantly elevated, and the highest expression was at a concentration of 1.6%. This could have been attributed to the fact that lysine and glutamate could activate myogenic factor expressions in vivo, promoting the development of skeletal muscles [20,21,22,23]. Cai et al. found that a lack of lysine in the diet could significantly reduce MyoD, mrf5 as well as MSTN1 transcriptions and reduce the growth performance of blunt snout bream [45]. In the study, we found that high expressions of LKB1 could promote skeletal muscle development and formation of flavor substances. From the perspective of LKB1 expression regulation, the 1.6% dipeptide diet was conducive for muscle growth and development in triploid crucian carp. This would help in optimization of feed formula. However, the specific regulatory mechanisms of lysine-Glutamate dipeptide on LKB1 should be evaluated further.

## 5. Conclusions

LKB1 could regulate skeletal muscle development and muscle integrity in triploid crucian carp. In this study, we cloned and analyzed LKB1 extracted from triploid crucian carp for the first time. Tissue expression analysis showed that LKB1 was widely expressed in tissues and reached the highest expression value in the brain, spleen and muscle. Short-term injection experiments showed that when LKB1 was activated or inhibited in a short time, expressions of MRFs and MSTN1 were coordinatively elevated or decreased. When LKB1 was activated, the content of IMP in muscle was significantly increased. The long-term injection experiment revealed that LKB1 activated for a long time can promote muscle development by expanding muscle fibers, and inhibited LKB1 inhibited can hinder muscle growth by shrinking muscle fibers and increasing the gap between muscle fibers. In addition, when LKB1 was activated for a long time, muscle texture profiles had some significant changes such as smaller hardness and adhesion, greater elasticity and chewiness. Analysis of the effect of different concentrations of lysine-glutamate dipeptide diet on LKB1 expression in triploid crucian carp showed that, at a dipeptide concentration of 1.6%, the expression of LKB1 was increased significantly. These data provide a basis for further study on the nutritional regulation and physiological function of LKB1 in fish.

## Figures and Tables

**Table 1:** The sequences of primers used in this study.

Primer Names	Sequence (5′→3′)	Use
LKB1-F	CGTTGCTTCGGAAAACTAAT	CDS
LKB1-R	CATAACGCTCACAGCTCCTC	CDS
RT-LKB1-F	ATCTCAGACCTGGGTGTAGCAG	qRT-PCR
RT-LKB1-R	GGCTCGTGGTTATGTTGTATAGTG	qRT-PCR
RT-actin-F	CTGCCCACCAACGATCTGTCCC	qRT-PCR
RT-actin-R	CTTATTTAGCCCCGCCCCCTCT	qRT-PCR
RT-MyoG-F	TCTTCGCAGACCAGCGTTTTT	qRT-PCR
RT-MyoG-R	CAACCCCACTCCGTTTGACAG	qRT-PCR
RT-MyoD-F	GAAAAACCACCAACGCTGACC	qRT-PCR
RT-MyoD-R	CAGGATCTCCACTTTGGGCAG	qRT-PCR
RT-Myf5-F	GTTTGAGGCACTACGGCG	qRT-PCR
RT-Myf5-R	CTTTCAGAACAGCTTGAGGAAG	qRT-PCR
RT-MRF4-F	GTCAGTGTCTAATGTGGGCTTG	qRT-PCR
RT-MRF4-R	GGATTGGGCACCGTCTTTTTCT	qRT-PCR
RT-MSTN1-F	GTTCTGGGGGATGACAGTAAGG	qRT-PCR
RT-MSTN1-R	TTGAACGATGGGGTCAGGCTCT	qRT-PCR

**Table 2:** Diet formulation and chemical composition of the lysine-glutamate dipeptide diets (% dry matter).

	Dietary Lysine-Glutamate Dipeptide Levels (%)					
	0.0	0.4	0.8	1.2	1.6	2.0
Fishmeal ^1^	12.00	12.00	12.00	12.00	12.00	12.00
Soybean meal ^1^	20.00	20.00	20.00	20.00	20.00	20.00
Rapeseed meal ^1^	15.00	15.00	15.00	15.00	15.00	15.00
Casein ^1^	6.50	6.50	6.50	6.50	6.50	6.50
Fish oil	3.00	3.00	3.00	3.00	3.00	3.00
Soybean oil	3.00	3.00	3.00	3.00	3.00	3.00
Cornstarch	16.80	16.80	16.80	16.80	16.80	16.80
Wheat flour	10.00	10.00	10.00	10.00	10.00	10.00
Choline	0.50	0.50	0.50	0.50	0.50	0.50
Premix ^2^	3.00	3.00	3.00	3.00	3.00	3.00
CMC	3.00	3.00	3.00	3.00	3.00	3.00
Cellulose	7.20	6.80	6.40	6.00	5.60	5.20
Lysine-glutamate ^3^	0.00	0.40	0.80	1.20	1.60	2.00
Total	100.00	100.00	100.00	100.00	100.00	100.00
Proximate composition						
Crude protein	32.01	32.41	32.81	33.21	33.61	34.01
Crude lipid	8.07	8.07	8.07	8.07	8.07	8.07
Moisture	10.05	12.31	9.80	9.70	11.18	0.93
Ash	6.78	6.48	6.97	6.54	6.77	7.15

^1^ All of these ingredients were purchased from Hunan Zhenghong Science and Technology Develop Co., Ltd., Changsha, China. ^2^ Vitamin and mineral premix (mg/kg diet): Vitamin B_12_, 0.02; folic acid, 5; calcium pantothenate, 50; inositol, 100; niacin, 100; biotin, 0.1; Vitamin B_1_, 20; Vitamin B_2_, 20; Vitamin B_6_, 20; Vitamin A, 11; Vitamin D, 2; Vitamin E, 50; Vitamin K, 10; Vitamin C, 100; cellulose, 3412; CaH_2_PO_4_·2H_2_O, 7650.6; FeSO_4_·7H_2_O, 2286.2; C_6_H_10_CaO_6_·5H_2_O, 1750.0; ZnSO_4_·7H_2_O, 178.0; NaCl, 500.0; MgSO_4_·7H_2_O, 8155.6; NaH_2_PO_4_·2H_2_O, 12,500.0; KH_2_PO_4_, 16,000.0; MnSO_4_·H_2_O, 61.4; CuSO_4_·5H_2_O, 15.5; CoSO_4_·7H_2_O, 0.91; KI, 1.5; Na_2_SeO_3_, 0.60; Corn starch, 899.7. ^3^ Lysine-glutamate: purchased from Shanghai Acmec Biochemical Co., Ltd., Shanghai, China.

## Data Availability

The data that support the findings of this study are available from the corresponding author upon reasonable request.
